# Implementation of a standardized Impella-specific management pathway in cardiogenic shock

**DOI:** 10.1186/s40560-026-00937-9

**Published:** 2026-09-10

**Authors:** Kenji Kanenawa, Akihiro Isotani, Kento Matsui, Keisuke Miyamoto, Katsunori Miyahara, Norihito Oyanagi, Kengo Korai, Toshinari Kaku, Shinichi Tsumaru, Takehiko Matsuo, Norimasa Matsuda, Shinichi Kakumoto, Takenori Domei, Makoto Hyodo, Shinichi Shirai, Kenji Ando, Koh Ono

**Affiliations:** 1 Department of Cardiology, Kokura Memorial Hospital, 3-2-1 Asano, Kokurakita-ku, Kitakyushu, Fukuoka 802-8555 Japan; 2 Department of Cardiovascular Surgery, Kokura Memorial Hospital, Kitakyushu, Fukuoka Japan; 3 Department of Anesthesiology and Intensive Care Medicine, Kokura Memorial Hospital, Kitakyushu, Fukuoka Japan; 4 Department of Cardiovascular Medicine, Kyoto University Graduate School of Medicine, Kyoto, Japan

**Keywords:** Cardiogenic shock, Impella, Mechanical circulatory support, Implementation, Intensive care

## Abstract

**Background:**

Device-related complications remain frequent during Impella support. Although coordinated multidisciplinary care and standardized protocols are recommended for cardiogenic shock, Impella-specific management pathways have not been established. We evaluated implementation of a standardized Impella-specific management pathway (SIM) and the accompanying changes in care processes and in-hospital outcomes.

**Methods:**

This single-center, before-and-after observational study included 190 consecutive patients with cardiogenic shock treated with Impella, 88 before implementation and 102 after SIM introduction. SIM combined multidisciplinary education and implementation support, peri-implant strategy, access-site and limb-perfusion management, hemolysis surveillance, and hemodynamic optimization. The primary endpoint was all-cause in-hospital mortality, and the key secondary endpoint was Impella-related Bleeding Academic Research Consortium type 3 or 5 bleeding. Both were analyzed using overlap-weighted cause-specific Cox models.

**Results:**

After SIM introduction, pre-PCI Impella use among patients with acute myocardial infarction increased from 36.2 to 76.2%, and 16-Fr sheath use increased from 0 to 80.2%. The rate of detected hemolysis increased progressively, whereas the time spent outside the target ranges for mean arterial pressure and central venous pressure decreased. After overlap weighting, all covariates included in the propensity-score model were balanced (absolute standardized mean difference <0.10). In-hospital death occurred in 58.0% before implementation and 33.3% after SIM introduction (hazard ratio, 0.51; 95% confidence interval, 0.32–0.84; *P* = 0.009). No significant interaction was observed according to SCAI shock stage, arrest or ECPELLA status at Impella insertion. Impella-related type 3 or 5 bleeding occurred in 30.7 and 12.7%, respectively (hazard ratio, 0.33; 95% confidence interval, 0.16–0.67; *P* = 0.002). BARC type 5 bleeding, acute limb ischemia, and stroke did not differ significantly.

**Conclusions:**

SIM was progressively implemented across the catheterization laboratory and ICU, accompanied by measurable changes in care processes and lower rates of major bleeding and in-hospital death. Prospective multicenter studies should assess whether these findings are reproducible.

**Supplementary Information:**

The online version contains supplementary material available at https://doi.org/10.1186/s40560-026-00937-9.

## Background

Cardiogenic shock (CS) remains associated with high mortality despite advances in reperfusion therapy, vasoactive treatment, and temporary mechanical circulatory support (MCS) [1–3]. Randomized trials of routine intra-aortic balloon pump support (IABP) and early venoarterial extracorporeal membrane oxygenation (ECMO) have not demonstrated a consistent survival benefit in acute myocardial infarction (AMI) with CS [4, 5]. In contrast, the DanGer Shock trial showed that Impella support reduced mortality in selected patients with ST-segment elevation myocardial infarction and CS [6].

The Impella microaxial flow pump unloads the left ventricle while augmenting forward flow, but device-related complications may offset this benefit. In the DanGer Shock trial and contemporary Impella trials such as STEMI-DTU and CHIP-BCIS3, bleeding, vascular complications, limb ischemia, hemolysis, and renal replacement therapy were frequent [7, 8]. Access-site bleeding is particularly important, with major bleeding or vascular complications reported in a substantial proportion of patients treated with Impella [9]. Japanese Registry for Percutaneous Ventricular Assist Device (J-PVAD) data also associate bleeding and ischemic complications with increased in-hospital mortality [10]. Hemolysis may result from the shear stress of axial-flow support or device malposition [11]. It may increase the risk of acute kidney injury, thromboembolic complications, and renal replacement therapy [12]. Recent expert recommendations identify coordinated multidisciplinary care and standardized protocols as central to cardiogenic shock management [13]. However, management during Impella support extends beyond the prevention of device-related complications. It also requires coordinated decisions regarding patient selection and timing of Impella support, hemodynamic optimization, reassessment of circulatory adequacy, and escalation to advanced mechanical circulatory support when needed [14–16]. At our institution, these elements were progressively refined and integrated into a standardized Impella-specific management pathway (SIM), supported by repeated multidisciplinary education and an electronic order set to promote consistent practice across the catheterization laboratory and ICU. An earlier descriptive correspondence from our institution reported selected complication-prevention practices and crude outcomes across three implementation phases in a restricted cohort [17]. That report focused primarily on the implementation of complication-prevention measures and was not designed to evaluate the broader SIM framework or its association with clinical outcomes. We therefore evaluated the implementation of SIM in an expanded all-comer cohort, examined changes in care processes as the pathway matured, and assessed the associations of SIM introduction with in-hospital mortality and device-related complications.

## Methods

### Study design and population

**Fig. 1:**
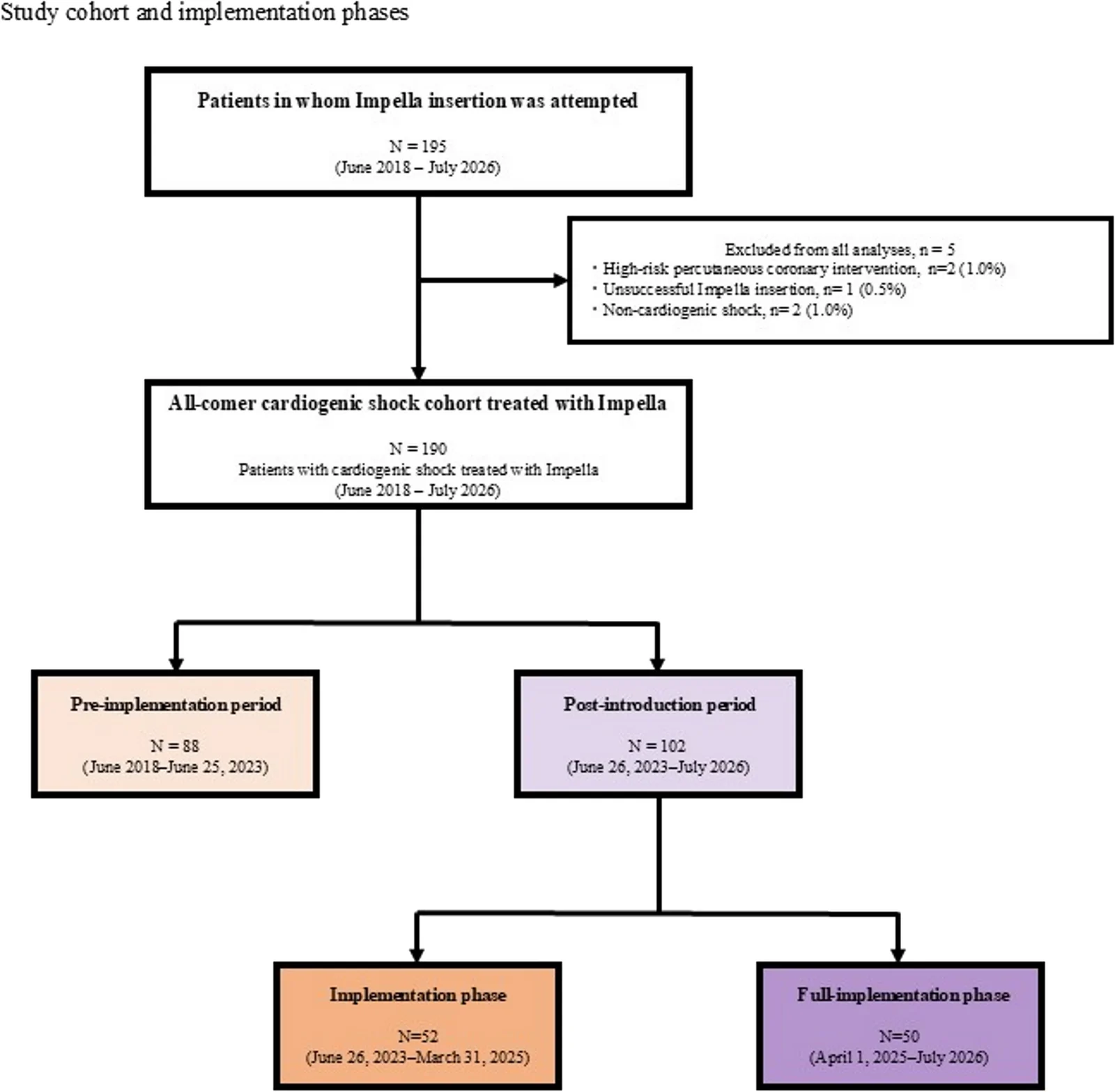
Study patient flow

The study flowchart is shown in Fig. 1. This single-center, before-and-after observational cohort included 190 consecutive patients with cardiogenic shock who underwent successful Impella insertion. All 159 patients included in our previous Critical Care correspondence were included in the present cohort. The present study additionally included 31 patients, comprising 21 patients who had been excluded from the previous restricted cohort because of atypical or extreme clinical presentations and 10 patients treated after the previous dataset cutoff [17]. Most study data were collected retrospectively. Data for patients treated after December 5, 2025, were collected under an institutional registry protocol approved on that date. The primary comparison was between the pre-implementation period (*n *= 88) and the post-introduction period (*n *= 102). SIM was implemented progressively rather than introduced as a completed bundle at a single time point. The post-introduction period began on June 26, 2023, when the access-site and limb-perfusion management strategy, including use of the 16-Fr non-peel-away sheath, and the multidisciplinary education and implementation infrastructure were initiated. On March 12, 2024, the peri-implant strategy and hemodynamic optimization protocol were formally disseminated through this educational framework. Quantitative plasma-free hemoglobin surveillance was introduced on April 14, 2024, and rollout of the Impella-specific electronic medical record order set began on January 18, 2025. Continuous lower-limb regional oxygen saturation monitoring was introduced on April 1, 2025, marking full implementation of the SIM pathway. The implementation and full-implementation phases were combined for the primary comparison because they represented consecutive stages of the same institutional implementation program. To describe implementation maturation, the post-introduction period was additionally divided into an implementation phase (*n *= 52; before April 1, 2025) and a full-implementation phase (*n *= 50; from April 1, 2025). Full implementation did not require every optional intervention to be used in every patient. The implementation dates and patient-level exposure and uptake of individual SIM components are summarized in Supplementary Table 1. The restricted sensitivity cohort excluded 22 atypical presentations, including postcardiotomy cardiogenic shock, procedure-related catastrophic collapse, mechanical complications after acute myocardial infarction, and extreme or prolonged low-flow states (*n *= 168; 73 pre-implementation and 95 post-introduction). The study protocol and opt-out approach were approved by the Institutional Review Board of Kokura Memorial Hospital (approval no. 25120501), which waived the requirement for written informed consent. An opt-out approach was used in accordance with guidance from the Japanese Ministry of Health, Labour and Welfare. The broader institutional registry was registered with the University Hospital Medical Information Network in May 2026 (UMIN000061600). This registration was not specific to the present SIM analysis and did not constitute a statistical analysis plan for this study (Table 1).

**Table 1:** Core processes and intended objectives of standardized Impella-specific management

Domain	Standardized processes and triggers	Management objective
Education and implementation infrastructure	Repeated multidisciplinary education and an Impella-specific electronic order set covering monitoring frequency, physiological targets, notification and reassessment triggers, management of suction or inadequate flow, and the potential roles of advanced MCS options, including Impella 5.5	Reduce practice variation, improve catheterization laboratory-to-ICU communication, facilitate early recognition of inadequate support, and promote timely consultation with clinicians experienced in advanced MCS
Peri-implant strategy	Structured assessment of Impella timing before PCI in selected patients with AMI-CS; systemic and lower-limb perfusion checkpoints before ICU transfer	Optimize support timing and identify inadequate circulatory or limb perfusion before ICU transfer
Access-site and limb-perfusion management	Preferred non–peel-away 16-Fr sheath strategy for femoral Impella CP support; limb-perfusion reassessment; continuous lower-limb rSO₂ surveillance with predefined reassessment triggers	Reduce access-site bleeding and detect limb hypoperfusion
Hemolysis surveillance and management	Routine quantitative plasma-free hemoglobin monitoring; protocolized response to suspected hemolysis	Detect hemolysis early and prompt device and hemodynamic reassessment
Hemodynamic optimization	MAP target 55–85 mmHg; CVP-guided congestion and preload management; physiology-guided assessment and selective RV/pulmonary vascular support	Maintain stable Impella support while avoiding excessive afterload and congestion

SIM standardized monitoring, physiological targets, reassessment, and the options to consider in each domain; it did not mandate uniform treatment. Decisions about pre-PCI Impella, milrinone or inhaled nitric oxide, and MCS escalation remained individualized

*AMI-CS* acute myocardial infarction-related cardiogenic shock, *CVP* central venous pressure, *ICU* intensive care unit, *MAP* mean arterial pressure, *MCS *mechanical circulatory support, *PCI *percutaneous coronary intervention, *rSO₂* regional oxygen saturation, *RV * right ventricular, *SIM *standardized Impella-specific management

### Standardized Impella-specific management

SIM standardized modifiable processes from Impella insertion in the catheterization laboratory through ICU care. These comprised education and implementation infrastructure [14, 18], peri-implant strategy [19–21], access-site and limb-perfusion management [22–24], hemolysis surveillance and management [25–27], and hemodynamic optimization [28, 29]. Operational details and the rationale for each component are provided in Supplementary Table 2.

The program was implemented through repeated multidisciplinary education and an Impella-specific electronic medical record order set. Educational sessions for physicians, critical care nurses, catheterization laboratory staff, and clinical engineers standardized monitoring targets, notification criteria, and reassessment processes. The sessions also addressed when Impella could be considered before PCI in patients with AMI-CS at high risk of hemodynamic collapse during the procedure. The order set specified hemodynamic targets, plasma-free hemoglobin surveillance and protocolized haptoglobin use, lower-limb regional oxygen-saturation monitoring, limb-perfusion reassessment, and triggers for reassessment of circulatory adequacy and consultation regarding possible MCS escalation. A non-peel-away 16-Fr sheath was adopted to reduce access-site leakage around the Impella repositioning sheath [22]. The protocol included haptoglobin for elevated plasma-free hemoglobin because of its hemoglobin-scavenging action and earlier reports of possible protection from hemolysis-associated organ injury [25, 26]. Before SIM implementation, red blood cell transfusion was generally considered when hemoglobin decreased below 10 g/dL as part of routine institutional practice. With SIM implementation, this threshold was explicitly incorporated into the standardized order set.

### Definitions

No formal institutional definition of cardiogenic shock based on prespecified numerical thresholds was established during the study period. Cardiogenic shock was diagnosed clinically by the attending physicians and treating cardiovascular team based on an integrated assessment of the clinical presentation, hemodynamic status, evidence of inadequate systemic perfusion, and the underlying cardiac condition. No fixed thresholds for blood pressure, lactate, or other individual variables were mandated for the diagnosis of cardiogenic shock or for Impella eligibility. The decision to initiate Impella support was made by the treating team according to the overall clinical context and anticipated need for circulatory support.

ECPELLA was defined as combined support with Impella and extracorporeal membrane oxygenation (ECMO), and ECPELLA at time zero was defined as initiation of both Impella and VA-ECMO support during the same catheterization laboratory session, irrespective of the sequence of device implantation. A brief arrhythmic event requiring a single defibrillation without impaired consciousness or ongoing resuscitation was not classified as cardiac arrest according to the Society for Cardiovascular Angiography and Interventions (SCAI) shock classification [30]. Advanced mechanical circulatory support was defined as Impella 5.0/5.5 or Impella support through alternative access, including the subclavian artery or direct ascending aorta. Bleeding events were classified according to Bleeding Academic Research Consortium (BARC) criteria [31]. Hemolysis events were defined as hematuria and/or elevated plasma-free hemoglobin.

### Outcomes

The primary comparison was between the pre-implementation and post-introduction periods. The primary endpoint was all-cause in-hospital mortality. The key secondary endpoint was Impella-related BARC type 3 or 5 bleeding. Events included in the Impella-related bleeding endpoint were additionally classified as BARC type 3a, 3b, or 5b according to severity. Other secondary outcomes included hemolysis events, acute limb ischemia during Impella support, stroke, newly initiated continuous hemodiafiltration, red blood cell transfusion, and changes in management-process measures. Impella insertion was time zero for all post-implant outcome and management-process analyses; only events occurring after insertion were included.

### Data sources

Trained medical administrative staff extracted patient characteristics, medical history, baseline covariates, management-process measures, and outcomes from the medical records using standardized definitions. The investigators reviewed data accuracy and completeness and resolved discrepancies by consensus after checking the source records.

### Statistical analysis

Continuous variables are reported as mean ± standard deviation or median [interquartile range] and were compared with Welch’s *t* test or the Wilcoxon rank-sum test, as appropriate. Categorical variables are reported as counts and percentages and were compared with Pearson’s *χ*^2^ test or Fisher’s exact test. For the descriptive three-phase analysis, overall comparisons used one-way analysis of variance or the Kruskal–Wallis test for continuous variables, as appropriate, and Pearson’s *χ*^2^ test or Fisher’s exact test for categorical variables; ordered trends were assessed with the Jonckheere–Terpstra test for continuous variables and the Cochran–Armitage test for categorical variables.

The propensity-score model included age, sex, systolic blood pressure, lactate, estimated glomerular filtration rate, total bilirubin, pre-Impella left ventricular ejection fraction, out-of-hospital cardiac arrest, in-hospital cardiac arrest, extracorporeal cardiopulmonary resuscitation, atrial fibrillation, maintenance hemodialysis, hemoglobin, platelet count, prior heart failure, prior percutaneous coronary intervention or coronary artery bypass grafting, shock etiology, and ECPELLA at time zero. The propensity score was defined as the estimated probability that a patient was treated during the post-introduction period. Patients in the post-introduction period received a weight of 1 minus the propensity score, and patients in the pre-implementation period received the propensity score [32]. Covariate balance was assessed with absolute standardized mean differences, with <0.10 considered acceptable.

For all-cause in-hospital death, patients were followed from Impella insertion until in-hospital death or discharge alive. The primary measure of association was the cause-specific hazard ratio for in-hospital death from an overlap-weighted Cox proportional hazards model with a robust sandwich variance estimator. In the cause-specific Cox model, discharge alive was treated as a competing event and patients were censored at discharge. As an exploratory adjusted analysis of the temporal pattern across the three implementation phases, propensity scores for membership in the pre-implementation, implementation, and full-implementation phases were estimated using multinomial logistic regression with the same baseline covariates as in the primary propensity-score model. Generalized overlap weights were then derived to balance these covariates across the three phases. Phase-specific overlap-weighted risks of in-hospital death were estimated. Unadjusted cumulative-incidence curves for in-hospital death were estimated with the Aalen–Johansen method. For Impella-related BARC type 3 or 5 bleeding, the main analysis used an overlap-weighted cause-specific Cox model with robust variance; death or discharge before bleeding was treated as censoring in the cause-specific analysis. Overlap-weighted Aalen–Johansen estimates treated death or discharge without prior BARC type 3 or 5 bleeding as competing events. In addition to hazard ratios, overlap-weighted absolute risks and risk differences were estimated for in-hospital mortality and Impella-related BARC type 3 or 5 bleeding. Ninety-five percent confidence intervals for absolute risks and risk differences were obtained by bootstrap resampling with re-estimation of the propensity score and overlap weights in each resample.

Thirty-day mortality was examined as a sensitivity outcome using the same overlap-weighting framework.

Separate sensitivity analyses of mortality were conducted using the restricted sensitivity cohort, excluding patients treated with Impella 2.5, and excluding patients in the implementation phase. Propensity scores and overlap weights were re-estimated within each sensitivity cohort. In an additional analysis, calendar time, defined as years from the beginning of the study period to Impella insertion, was included as a continuous covariate in the overlap-weighted cause-specific Cox model. Secondary, three-phase, and exploratory *P* values were descriptive and were not adjusted for multiple comparisons. Analyses were performed with R version 4.6.0 and JMP version 14.3.0.

## Results

The primary all-comer cohort included 190 patients: 88 in the pre-implementation period and 102 in the post-introduction period. SCAI stage E was present in 48 of 88 patients (54.5%) and 61 of 102 (59.8%), respectively; acute myocardial infarction accounted for 47 of 88 (53.4%) and 64 of 102 (62.7%), respectively. Before weighting, the largest imbalances involved prior PCI or CABG, shock etiology, platelet count, maintenance hemodialysis, atrial fibrillation, and ECPELLA at Impella insertion (Table 2). After overlap weighting, all propensity-score covariates had absolute standardized mean differences <0.10 (Supplementary Figure 1). Baseline characteristics across the three implementation phases are shown in Supplementary Table 3.

**Table 2:** Baseline characteristics before and after overlap weighting

Characteristic	Before overlap weighting			After overlap weighting		
	Pre-implementation period (*n *= 88)	Post-introduction period (*n *= 102)	|SMD|	Pre-implementation period, OW	Post-introduction period, OW	|SMD|
Demographics						
Age, years	68.8 [58.2, 76.8]	73.0 [61.8, 78.0]	0.15	69.5 [55.1, 78.4]	69.6 [57.5, 76.4]	<0.001
Male sex, *n* (%)	67 (76.1)	81 (79.4)	0.08	79.3%	79.3%	<0.001
Medical history						
Prior heart failure, *n* (%)	24 (27.3)	22 (21.6)	0.13	23.2%	23.2%	<0.001
Atrial fibrillation, *n* (%)	21 (23.9)	13 (12.7)	0.29	17.4%	17.4%	<0.001
Maintenance hemodialysis, *n* (%)	14 (15.9)	6 (5.9)	0.33	9.5%	9.5%	<0.001
Prior PCI or CABG, *n* (%)	28 (31.8)	10 (9.8)	0.56	16.6%	16.6%	<0.001
Shock etiology, *n* (%)						
Acute myocardial infarction	47 (53.4)	64 (62.7)	0.19	58.9%	58.9%	<0.001
Fulminant myocarditis	11 (12.5)	15 (14.7)	0.06	14.4%	14.4%	<0.001
Heart failure/arrhythmia	15 (17.0)	19 (18.6)	0.04	19.4%	19.4%	<0.001
PCCS/procedure-related/mechanical	15 (17.0)	4 (3.9)	0.44	7.2%	7.2%	<0.001
Presentation and shock severity						
Out-of-hospital cardiac arrest, *n* (%)	12 (13.6)	21 (20.6)	0.19	17.6%	17.6%	<0.001
In-hospital cardiac arrest, *n* (%)	26 (29.5)	23 (22.5)	0.16	26.0%	26.0%	<0.001
ECPR, *n* (%)	22 (25.0)	21 (20.6)	0.11	22.3%	22.3%	<0.001
SCAI stage C, *n* (%)	15 (17.0)	19 (18.6)	0.04	18.8%	18.7%	0.003
SCAI stage D, *n* (%)	25 (28.4)	22 (21.6)	0.16	27.1%	24.3%	0.06
SCAI stage E, *n* (%)	48 (54.5)	61 (59.8)	0.11	54.1%	57.0%	0.056
Systolic blood pressure, mmHg	73.2 ± 50.5	75.9 ± 51.6	0.05	77.9 ± 50.6	77.9 ± 52.6	<0.001
Lactate, mmol/L	6.0 ± 3.9	6.7 ± 4.5	0.17	6.5 ± 4.0	6.5 ± 4.5	<0.001
eGFR, mL/min/1.73 m^2^	40.5 ± 25.0	42.6 ± 22.8	0.09	43.1 ± 23.8	43.1 ± 23.5	<0.001
Total bilirubin, mg/dL	1.0 ± 0.7	0.9 ± 0.7	0.06	0.9 ± 0.7	0.9 ± 0.6	<0.001
Pre-Impella LVEF, %	22.7 ± 11.8	23.8 ± 12.8	0.09	23.2 ± 12.1	23.2 ± 11.9	<0.001
Hemoglobin, g/dL	12.3 ± 2.3	12.3 ± 2.4	0.01	12.4 ± 2.3	12.4 ± 2.4	<0.001
Platelet count, 10^4^/µL	20.6 ± 9.9	25.3 ± 14.1	0.39	22.7 ± 10.2	22.7 ± 11.6	<0.001
Mechanical circulatory support at time zero						
ECPELLA at Impella insertion, *n* (%)	55 (62.5)	45 (44.1)	0.38	51.5%	51.5%	<0.001

Values are presented as mean ± standard deviation, median [interquartile range], or *n* (%). Absolute standardized mean differences (|SMD|) assess balance; <0.10 was considered acceptable

ECPELLA at time zero was defined as initiation of both Impella and VA-ECMO support during the same catheterization laboratory session, irrespective of the sequence of device implantation

*CABG *coronary artery bypass grafting, *ECPR* extracorporeal cardiopulmonary resuscitation, *eGFR* estimated glomerular filtration rate, *LVEF* left ventricular ejection fraction, *OW* overlap weighting, *PCI* percutaneous coronary intervention, *SCAI* Society for Cardiovascular Angiography and Interventions, *SMD* standardized mean difference, *PCCS* postcardiotomy cardiogenic shock

### Changes in SIM-related management processes

Changes in SIM-related process measures and concurrent management practices are summarized in Table 3. Among patients with AMI who underwent PCI, Impella insertion before PCI increased from 17 of 47 (36.2%) to 48 of 63 (76.2%) (*P* < 0.001). Use of a 16-Fr femoral sheath increased from 0 of 87 to 77 of 96 (80.2%) (*P* < 0.001), and use of advanced mechanical circulatory support increased from 5 of 88 (5.7%) to 15 of 102 (14.7%) (*P *= 0.04). Swan–Ganz catheter use did not differ significantly. During the first 72 h after Impella insertion, total time outside the MAP target decreased from 14.8 ± 17.6 to 8.8 ± 13.5 h (*P *= 0.02), and cumulative time with CVP ≥10 mmHg decreased from 30.2 ± 26.2 to 17.4 ± 20.4 h (*P *= 0.003). Cumulative lactate burden and total fluid balance did not differ significantly. Use of inhaled nitric oxide (21.6 vs. 38.2%; *P *= 0.01) and haptoglobin (4.5 vs. 36.3%; *P* < 0.001) increased; PDE3 inhibitor use was not significantly different in the two-group comparison (23.9 vs. 36.3%; *P *= 0.06). In the three-phase analysis, pre-PCI Impella insertion, 16-Fr sheath use, advanced mechanical circulatory support, MAP-target control, CVP burden, inhaled nitric oxide, haptoglobin, and PDE3 inhibitor use showed significant ordered trends, whereas cumulative lactate burden and total fluid balance did not. To further examine the influence of early death, we repeated the descriptive process analysis among the 159 patients who survived beyond 72 h. The findings were similar to the primary available-case analysis. Time outside the MAP target remained lower after SIM introduction (14.6 ± 17.7 vs. 8.5 ± 13.3 h, *P *= 0.021), as did cumulative time with CVP ≥10 mmHg (30.1 ± 26.4 vs. 16.7 ± 19.6 h, *P *= 0.002).

**Table 3:** Changes in SIM-related management processes and clinical practice before and after SIM introduction

Characteristic	Two-group comparison			Three-phase analysis			
	Pre-implementation period (*N *= 88)	Post-introduction period (*N *= 102)	*P* value	Implementation phase (*N *= 52)	Full-implementation phase (*N *= 50)	Overall *P* value	*P* for trend
Catheterization laboratory and device strategy							
Impella insertion before PCI, *n*/*N* (%)	17/47 (36.2%)	48/63 (76.2%)	<0.001	25/37 (67.6%)	23/26 (88.5%)	<0.001	<0.001
Impella insertion after PCI, *n*/*N* (%)	16/47 (34.0%)	6/63 (9.5%)	0.001	4/37 (10.8%)	2/26 (7.7%)	0.006	0.003
Impella insertion after ICU admission, *n*/*N* (%)	14/47 (29.8%)	9/63 (14.3%)	0.048	8/37 (21.6%)	1/26 (3.8%)	0.03	0.011
IABP-to-Impella escalation, *n*/*N* (%)	24/88 (27.3%)	12/102 (11.8%)	0.007	11/52 (21.2%)	1/50 (2.0%)	0.001	<0.001
Use of a 16-Fr femoral sheath, *n*/*N* (%)	0/87 (0.0%)	77/96 (80.2%)	<0.001	33/50 (66.0%)	44/46 (95.7%)	<0.001	<0.001
Advanced MCS, *n*/*N* (%)	5/88 (5.7%)	15/102 (14.7%)	0.04	4/52 (7.7%)	11/50 (22.0%)	0.008	0.004
Swan–Ganz catheter, *n*/*N* (%)	82/88 (93.2%)	94/102 (92.2%)	0.79	50/52 (96.2%)	44/50 (88.0%)	0.28	0.35
Hemodynamic management during the first 72 h							
Time outside MAP target, 0–24 h	6.4 ± 8.5 (*n *= 83)	3.2 ± 5.3 (*n *= 91)	0.004	5.2 ± 6.6 (*n *= 46)	1.1 ± 2.1 (*n *= 45)	<0.001	<0.001
Time outside MAP target, 24–48 h	4.7 ± 7.7 (*n *= 77)	2.9 ± 5.9 (*n *= 89)	0.10	4.7 ± 7.4 (*n *= 44)	1.2 ± 3.2 (*n *= 45)	0.002	0.007
Time outside MAP target, 48–72 h	3.8 ± 6.6 (*n *= 72)	2.6 ± 5.9 (*n *= 87)	0.23	4.4 ± 7.8 (*n *= 43)	0.8 ± 2.0 (*n *= 44)	0.006	0.004
Total time outside MAP target, 0–72 h	14.8 ± 17.6 (*n *= 67)	8.8 ± 13.5 (*n *= 86)	0.02	14.7 ± 16.6 (*n *= 42)	3.2 ± 5.9 (*n *= 44)	<0.001	<0.001
Cumulative duration of lactate >2 mmol/L, 0–24 h	13.3 ± 10.3 (*n *= 84)	13.0 ± 9.9 (*n *= 93)	0.81	13.9 ± 10.1 (*n *= 48)	11.9 ± 9.7 (*n *= 45)	0.57	0.47
Cumulative duration of lactate >2 mmol/L, 24–48 h	8.0 ± 10.6 (*n *= 76)	5.8 ± 9.1 (*n *= 89)	0.17	6.9 ± 9.8 (*n *= 44)	4.8 ± 8.4 (*n *= 45)	0.47	0.26
Cumulative duration of lactate >2 mmol/L, 48–72 h	4.2 ± 8.6 (*n *= 72)	3.5 ± 8.0 (*n *= 87)	0.56	4.1 ± 8.9 (*n *= 43)	2.8 ± 7.1 (*n *= 44)	0.79	0.50
Cumulative duration of lactate >2 mmol/L, 0–72 h	23.5 ± 26.6 (*n *= 68)	21.7 ± 22.8 (*n *= 87)	0.66	23.5 ± 24.5 (*n *= 43)	19.9 ± 21.2 (*n *= 44)	0.90	0.77
Fluid balance, 0–24 h, mL	1840 ± 1800 (*n *= 84)	1435 ± 1414 (*n *= 92)	0.10	1471 ± 1316 (*n *= 47)	1397 ± 1523 (*n *= 45)	0.56	0.26
Fluid balance, 24–48 h, mL	1532 ± 1541 (*n *= 74)	1622 ± 1667 (*n *= 89)	0.72	2246 ± 1886 (*n *= 44)	1012 ± 1145 (*n *= 45)	<0.001	0.09
Fluid balance, 48–72 h, mL	1227 ± 1053 (*n *= 69)	1151 ± 1142 (*n *= 87)	0.67	1501 ± 1276 (*n *= 43)	809 ± 880 (*n *= 44)	0.02	0.07
Total fluid balance, 0–72 h, mL	4605 ± 3609 (*n *= 66)	4131 ± 3166 (*n *= 86)	0.40	5056 ± 3414 (*n *= 42)	3247 ± 2658 (*n *= 44)	0.01	0.12
Cumulative duration of CVP ≥10 mmHg, 0–24 h	8.8 ± 10.2 (*n *= 68)	7.0 ± 9.0 (*n *= 87)	0.27	9.6 ± 9.9 (*n *= 45)	4.3 ± 7.1 (*n *= 42)	0.03	0.11
Cumulative duration of CVP ≥10 mmHg, 24–48 h	11.0 ± 10.3 (*n *= 61)	5.8 ± 7.8 (*n *= 84)	0.001	7.6 ± 9.1 (*n *= 42)	4.0 ± 5.8 (*n *= 42)	0.004	<0.001
Cumulative duration of CVP ≥10 mmHg, 48–72 h	11.9 ± 10.4 (*n *= 62)	4.7 ± 7.6 (*n *= 81)	<0.001	6.7 ± 9.3 (*n *= 40)	2.8 ± 5.0 (*n *= 41)	<0.001	<0.001
Cumulative duration of CVP ≥10 mmHg, 0–72 h	30.2 ± 26.2 (*n *= 53)	17.4 ± 20.4 (*n *= 82)	0.003	24.1 ± 24.0 (*n *= 40)	11.0 ± 13.8 (*n *= 42)	<0.001	<0.001
Pharmacologic adjuncts and supportive care							
Inhaled nitric oxide, *n*/*N* (%)	19/88 (21.6%)	39/102 (38.2%)	0.01	20/52 (38.5%)	19/50 (38.0%)	0.046	0.03
Haptoglobin, *n*/*N* (%)	4/88 (4.5%)	37/102 (36.3%)	<0.001	14/52 (26.9%)	23/50 (46.0%)	<0.001	<0.001
PDE3 inhibitor, *n*/*N* (%)	21/88 (23.9%)	37/102 (36.3%)	0.06	10/52 (19.2%)	27/50 (54.0%)	<0.001	<0.001

Values are mean ± standard deviation (available *n*) or *n*/available *N* (%)

^a^Timing categories were mutually exclusive and were available for 110 patients with AMI who underwent PCI. Management-process variables were not included in the baseline propensity-score model because they occurred after exposure assignment

Post-implant 72-h measures are available-case summaries and may depend on survival, device duration, and monitoring. *P* values are descriptive without multiplicity adjustment

*CVP* central venous pressure, *IABP* intra-aortic balloon pump, *MAP* mean arterial pressure, *MCS* mechanical circulatory support, *PCI* percutaneous coronary intervention

### In-hospital mortality

**Fig. 2:**
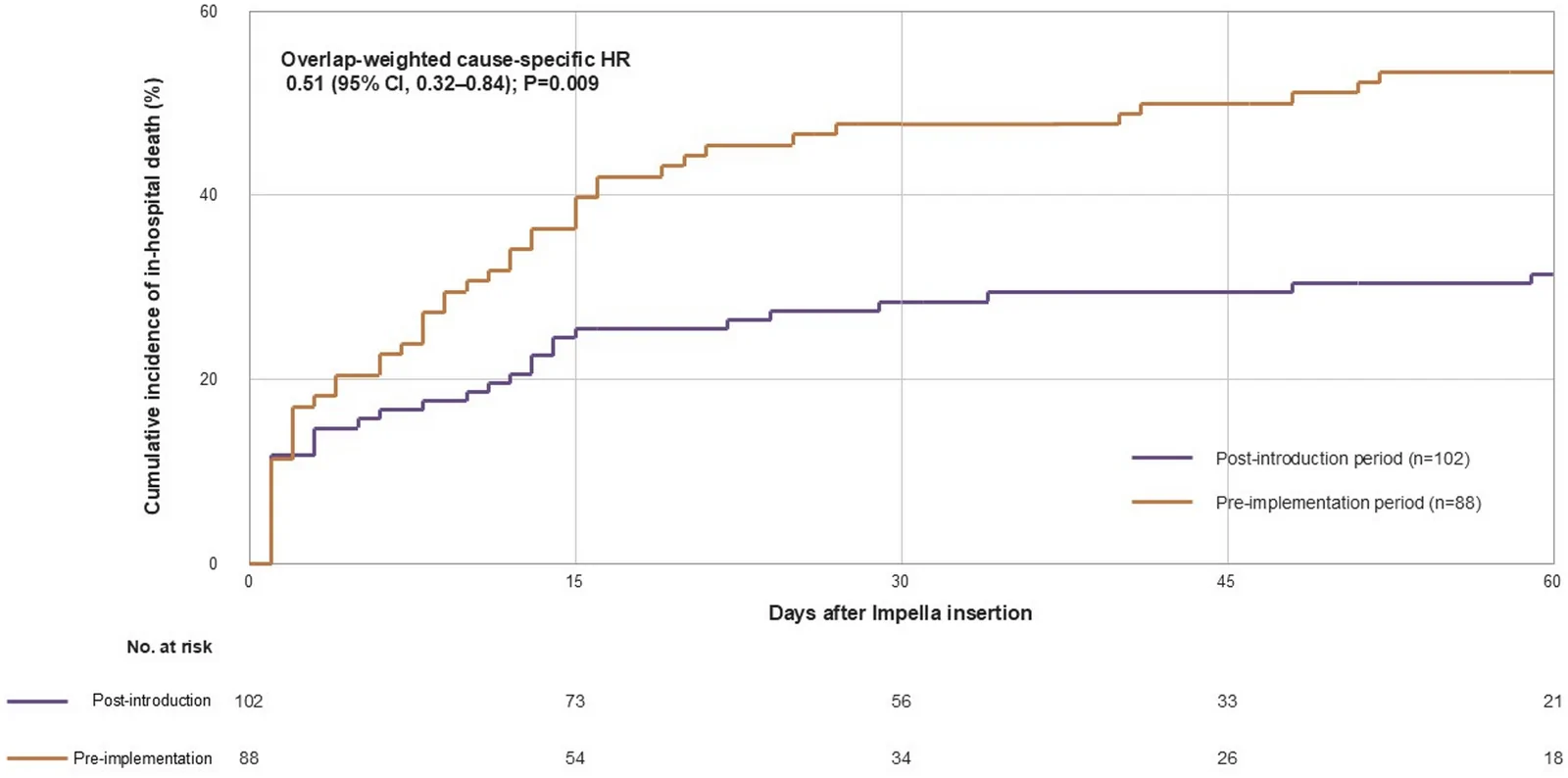
Unadjusted Aalen–Johansen cumulative-incidence curves for all-cause in-hospital death after Impella insertion. The overlap-weighted cause-specific hazard ratio from the primary Cox model is shown in the figure. Curves are displayed through 60 days after Impella insertion. *CI* confidence interval, *HR* hazard ratio.

**Fig. 3:**
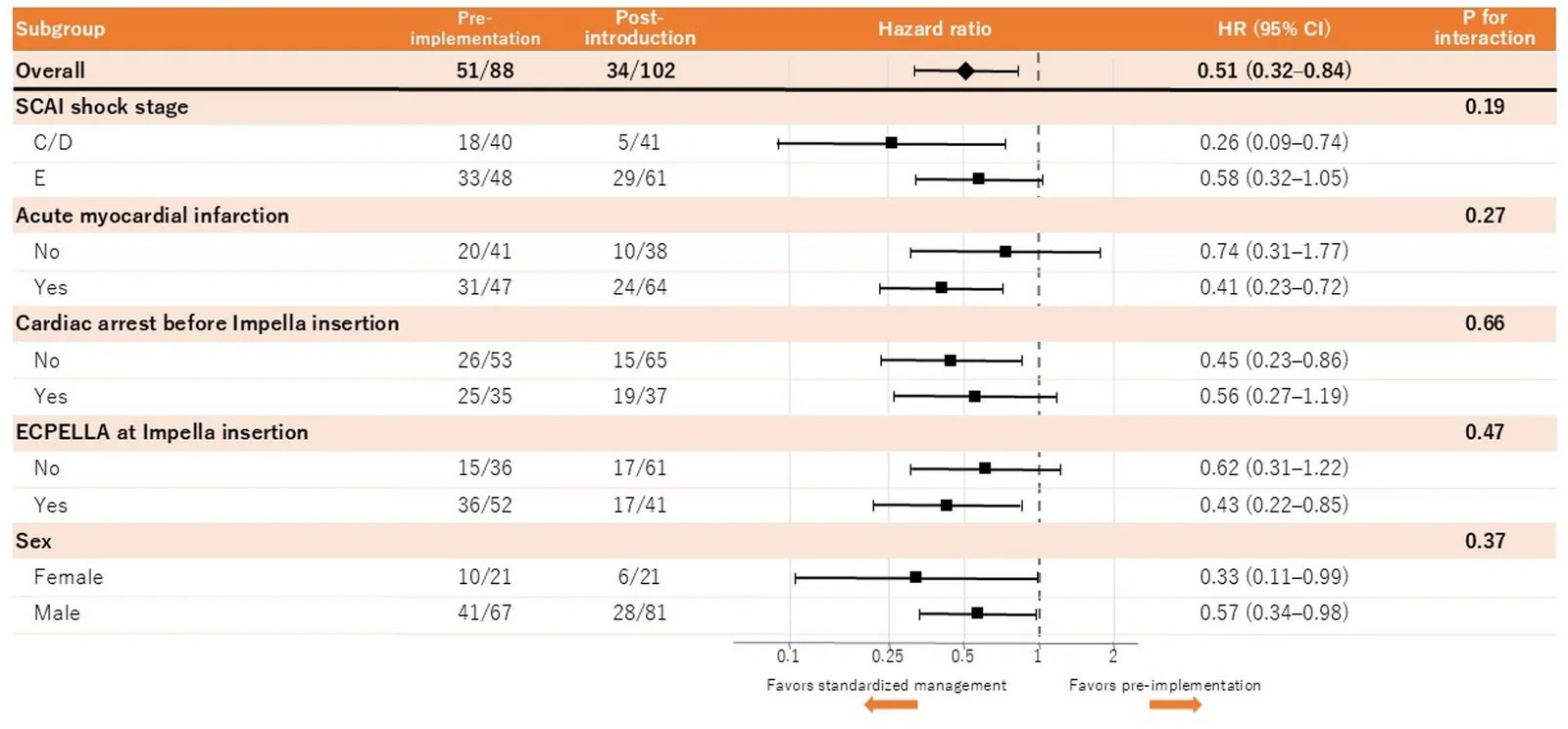
Subgroup analysis of all-cause in-hospital mortality. Separate overlap-weighted cause-specific Cox models with robust sandwich variance estimators were fitted in the full cohort for each subgroup variable. Each model included study period, the subgroup variable, and their interaction. Primary-analysis overlap weights were retained without re-estimation. *P* values for interaction were obtained from the interaction terms

In-hospital death occurred in 51 of 88 patients (58.0%) in the pre-implementation period and 34 of 102 (33.3%) in the post-introduction period (*P* < 0.001). In the primary overlap-weighted cause-specific Cox model, the post-introduction period was significantly associated with a lower hazard of in-hospital death (HR, 0.51; 95% CI, 0.32–0.84; *P *= 0.009). Unadjusted Aalen–Johansen cumulative-incidence curves are shown in Fig. 2. In the three-phase analysis, in-hospital mortality was 58.0% (51/88) in the pre-implementation phase, 34.6% (18/52) during the implementation phase, and 32.0% (16/50) during the full-implementation phase (*P* for trend = 0.002). In the three-phase generalized overlap-weighted analysis, the estimated risks of in-hospital death were 58.7, 37.7, and 30.9%, respectively, with an overall decreasing trend across the three phases after weighting (*P* for trend = 0.02). In subgroup analyses, no significant interactions were observed across subgroups defined by SCAI shock stage, AMI status, cardiac arrest before Impella insertion, ECPELLA support, or sex (Fig. 3).

The overlap-weighted estimated risk of in-hospital death was 56.6% (95% CI, 45.3–67.6%) before implementation and 31.7% (95% CI, 22.5–41.4%) after SIM introduction, corresponding to a risk difference of −24.9 percentage points (95% CI, −38.1 to −11.2). The proportional hazards assumption was not violated (*P *= 0.717). Thirty-day mortality occurred in 42 of 88 patients (47.7%) before implementation and 29 of 102 patients (28.4%) after SIM introduction. The overlap-weighted risks were 45.8% (95% CI, 34.7–56.2%) and 26.6% (95% CI, 17.5–35.8%), respectively, with a risk difference of −19.3 percentage points (95% CI, −31.8 to −5.9).

Similar estimates were obtained in the restricted sensitivity cohort (HR, 0.52; 95% CI, 0.32–0.84; *P *= 0.008), after exclusion of patients treated with Impella 2.5 (HR, 0.47; 95% CI, 0.30–0.76; *P *= 0.002), after exclusion of the implementation phase (HR, 0.48; 95% CI, 0.25–0.93; *P *= 0.03), and after additional adjustment for calendar time (HR, 0.40; 95% CI, 0.17–0.95; *P *= 0.04) (Supplementary Table 4). Calendar-year trends in clinical outcomes are shown in Supplementary Table 5. In-hospital mortality did not decline monotonically during the pre-implementation years and was 54.5, 43.8, 66.7, 58.3, and 70.0% from 2018 to 2022, respectively.

### Clinical outcomes and major complications

**Fig. 4:**
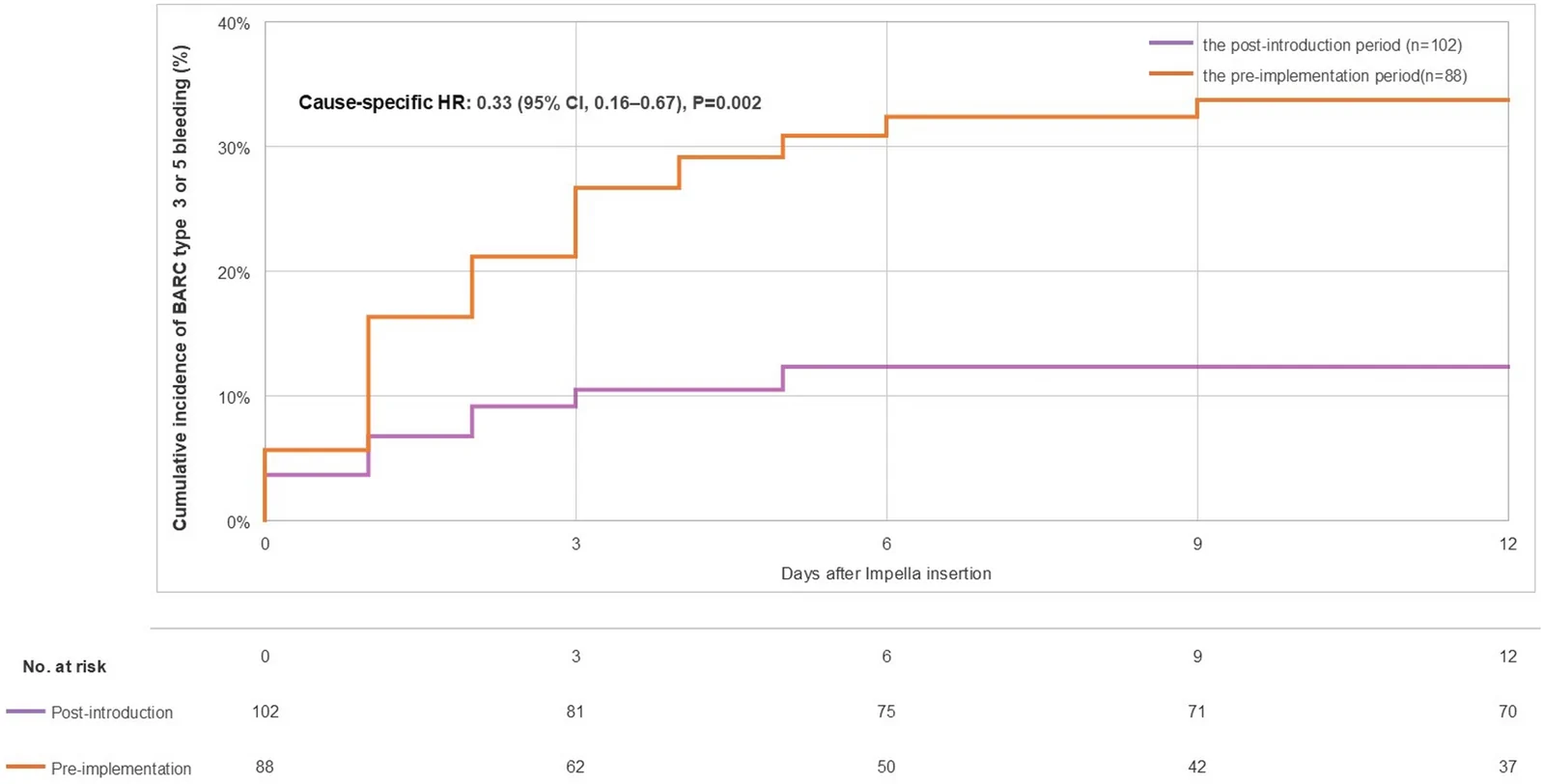
Cumulative incidence of Impella-related BARC type 3 or 5 bleeding. Aalen–Johansen cumulative-incidence curves for Impella-related BARC type 3 or 5 bleeding after Impella insertion according to study period. In-hospital death was treated as a competing event. *BARC* Bleeding Academic Research Consortium

Impella-related BARC type 3 or 5 bleeding occurred in 27 of 88 patients (30.7%) in the pre-implementation period and 13 of 102 (12.7%) in the post-introduction period (*P *= 0.004; Table 4). Among the 40 events, 14 were classified as BARC type 3a, 20 as type 3b, and 6 as type 5b. In the primary overlap-weighted cause-specific Cox model, the post-introduction period was associated with a lower cause-specific hazard of BARC type 3 or 5 bleeding (HR, 0.33; 95% CI, 0.16–0.67; *P *= 0.002). Unadjusted Aalen–Johansen cumulative-incidence curves are shown in Fig. 4. The overlap-weighted absolute risks were 33.5% (95% CI, 23.3–44.4%) and 12.4% (95% CI, 5.9–20.1%), respectively, corresponding to a risk difference of −21.1 percentage points (95% CI, −34.0 to −9.2). The proportional hazards assumption was not violated (*P *= 0.277). BARC type 3 or 5 bleeding declined across the three phases (30.7, 15.4 and 10.0%; overall *P *= 0.009; *P* for trend = 0.003). BARC type 5 bleeding, acute limb ischemia during Impella support, and stroke did not differ significantly. Hemolysis events, defined as hematuria and/or elevated plasma-free hemoglobin, were detected in 30.7 and 45.1% (*P *= 0.052), with an increasing trend across phases (*P* for trend = 0.01). Newly initiated continuous hemodiafiltration was required in 44.3 and 30.9% (*P *= 0.10), with a decline across the three phases (44.3, 35.4, and 26.1%; overall *P *= 0.14; *P* for trend = 0.04). Median red blood cell transfusion within 7 days decreased from 15 [8–32.5] to 12 [4–20] units (*P *= 0.009), and total transfusion decreased from 23 [10–52] to 16 [4–24] units (*P *= 0.001).

**Table 4:** Clinical outcomes and major complications

Event	Two-group comparison			Three-phase analysis			
	Pre-implementation period (*N *= 88)	Post-introduction period (*N *= 102)	*P* value	Implementation phase (*N *= 52)	Full-implementation phase (*N *= 50)	Overall *P* value	*P* for trend
Primary endpoint							
In-hospital mortality, *n*/*N* (%)	51/88 (58.0%)	34/102 (33.3%)	<0.001	18/52 (34.6%)	16/50 (32.0%)	0.003	0.002
Key secondary endpoint							
Impella-related BARC type 3 or 5 bleeding, n/*N* (%)	27/88 (30.7%)	13/102 (12.7%)	0.004	8/52 (15.4%)	5/50 (10.0%)	0.009	0.003
Impella-related BARC type 3a bleeding, *n*/*N* (%)	8/88 (9.1%)	6/102 (5.9%)		5/52 (9.6%)	1/50 (2.0%)		
Impella-related BARC type 3b bleeding, *n*/*N* (%)	16/88 (18.2%)	4 /102 (3.9%)		1/52 (1.9%)	3/50 (6.0%)		
Impella-related BARC type 5 bleeding, *n*/*N* (%)	3/88 (3.4%)	3/102 (3.0%)	1.00	2/52 (3.8%)	1/50 (2.0%)	1.000	0.70
Other complications and resource use							
Hemolysis event, *n*/*N* (%)	27/88 (30.7%)	46/102 (45.1%)	0.052	20/52 (38.5%)	26/50 (52.0%)	0.049	0.01
Acute limb ischemia during Impella support, *n*/*N* (%)	3/88 (3.4%)	4/102 (3.9%)	1.00	1/52 (1.9%)	3/50 (6.0%)	0.62	0.52
Stroke, *n*/*N* (%)	7/88 (8.0%)	9/102 (8.8%)	1.00	3/52 (5.8%)	6/50 (12.0%)	0.53	0.49
Newly initiated CHDF, *n*/*N* (%)	31/70 (44.3%)	29/94 (30.9%)	0.10	17/48 (35.4%)	12/46 (26.1%)	0.14	0.04
RBC transfusion within 7 days, units	15 [8–32.5]	12 [4–20]	0.009	9 [2–20.5]	15 [4–20]	0.019	0.047
Total RBC transfusion, units	23 [10–52]	16 [4–24]	0.001	14 [4–25]	17 [6–24]	0.006	0.006

Values are *n*/available *N* (%) or median [interquartile range]. Categorical two-group comparisons used Fisher’s exact test; three-phase overall comparisons used the Fisher–Freeman–Halton exact test, and ordered trends used the Cochran–Armitage test. Continuous variables used the Wilcoxon rank-sum, Kruskal–Wallis, and Jonckheere–Terpstra tests. All *P* values shown in this table are unadjusted. Secondary and three-phase *P* values are descriptive and were not adjusted for multiple comparisons

*BARC* Bleeding Academic Research Consortium, *CHDF* continuous hemodiafiltration, *RBC *red blood cell

## Discussion

SIM was progressively implemented across the catheterization laboratory and ICU, with marked changes in device timing, access management, complication surveillance, and hemodynamic care. These process changes were accompanied by lower rates of Impella-related BARC type 3 or 5 bleeding and in-hospital death. Similar mortality estimates were observed in sensitivity analyses, and no significant heterogeneity was detected across the examined clinical subgroups. The pathway standardized monitoring, reassessment, and trigger-based responses while leaving treatment decisions individualized. The findings therefore describe implementation of a multicomponent care pathway and its associated outcomes, not the effect of any single intervention. Notably, much of the reduction in mortality was already apparent during the implementation phase. The three-phase generalized overlap-weighted analysis showed a similar overall temporal pattern after adjustment for measured baseline differences, with estimated mortality risks of 58.7, 37.7, and 30.9% across the pre-implementation, implementation, and full-implementation phases, respectively (*P* for trend = 0.02). Several clinically important changes had already been introduced as part of SIM during the implementation phase. These included multidisciplinary education, standardized access-site and limb-perfusion management, and a peri-implant strategy emphasizing earlier Impella support in selected high-risk patients and pre-PCI insertion when feasible. Thus, the early mortality reduction occurred after SIM-related changes had begun and should not be interpreted as preceding SIM implementation. However, although mortality was numerically lower after full implementation, the largest reduction occurred during the implementation phase. The observed mortality difference therefore cannot be attributed specifically to completion of the full SIM bundle.

Major bleeding remains common during Impella support and is associated with poor outcomes in contemporary trials and registries [6–8, 10]. In this study, the hazard of BARC type 3 or 5 bleeding was lower after implementation in the overlap-weighted analysis, with a similar pattern in the cumulative-incidence curves. BARC type 5 bleeding and acute limb ischemia did not differ significantly. The reduction in major bleeding coincided with the 16-Fr sheath strategy and broader access-site surveillance. The higher frequency of the composite hemolysis event after implementation may reflect more intensive plasma-free hemoglobin surveillance rather than a biological increase. The small number of limb-ischemia events limits conclusions about this complication.

The most marked practice changes involved the 16-Fr sheath strategy, complication surveillance, and MAP and CVP management. Pre-PCI Impella use and adjunctive therapies also changed after multidisciplinary education and introduction of the order set, which defined when these options should be considered without mandating their use. Use of advanced MCS also increased during the study period. SIM specified early reassessment and expert consultation when support was inadequate but did not prescribe a particular MCS configuration. Similar team-based shock protocols and standardized approaches to Impella care have been reported previously [14–16, 18]. The 72-h hemodynamic variables were post-exposure, available-case measures affected by early death, device-removal timing, and monitoring intensity; they should not be interpreted as mediators or adjusted causal effects. Lactate burden and fluid balance did not differ significantly, so the process data did not show improvement across all measured physiological domains.

Newly initiated continuous hemodiafiltration and transfusion requirements were lower after implementation, but both findings should be interpreted cautiously. In particular, renal-replacement decisions are influenced by shock severity, duration of hypoperfusion, congestion, hemolysis, fluid accumulation, institutional thresholds, and survival time [29]. Lower CVP burden or more frequent haptoglobin administration could have contributed to the lower use of renal replacement therapy, but mediation was not tested and total fluid balance did not differ significantly.

Previous reports have described shock teams and protocol-based care, whereas this pathway focused specifically on Impella management from insertion through ICU care and complication surveillance [13, 14, 16, 20]. The progressive changes across implementation phases were consistent with changes in clinical practice following program implementation, although causality cannot be established. Prospective multicenter studies should assess reproducibility, implementation fidelity, and which components are most closely associated with outcomes.

### Limitations

First, the single-center before-and-after design precludes causal inference and remains vulnerable to residual temporal confounding. Although no separate major change in ICU organization or cardiogenic shock management outside SIM occurred around the transition to the implementation phase, institutional experience accumulated over time and cannot be fully separated from implementation of the pathway. SIM itself also incorporated changes in patient selection, timing of Impella support, and MCS management informed by accumulated institutional experience and previous evidence. In addition, because no formal institutional definition of cardiogenic shock based on fixed numerical thresholds was used during the study period, the diagnosis of cardiogenic shock and Impella candidacy were based on team-based clinical judgment, and temporal changes in case selection or treatment thresholds cannot be completely excluded. Overlap weighting addresses only measured baseline covariates and cannot eliminate these sources of confounding. Although the association with lower in-hospital mortality remained after additional adjustment for calendar time and after exclusion of the implementation phase, and the descriptive calendar-year analysis did not show a monotonic decline in mortality during the pre-implementation years, these findings do not establish that the observed improvement was attributable to SIM itself. Institutional learning, evolving operator and ICU experience, and other concurrent changes in care may have contributed to the observed temporal differences and remain partly inseparable from the effects of the standardized pathway. Second, implementation matured gradually and combined multiple components, so associations cannot be attributed to any individual element. Third, the 72-h process measures were based on available observations. Early death or device removal could shorten the observation period. Although routine monitoring frequency did not change with SIM implementation, measurement availability may have varied according to clinical severity and course, particularly in patients who died early or were critically ill. Missingness was therefore potentially informative, particularly for hemodynamic measures, and these process measures should be interpreted descriptively. Finally, the findings may not generalize to centers with different populations, volumes, resources, or Impella practices.

## Conclusions

Implementation of SIM was accompanied by measurable changes in catheterization laboratory and ICU care and by lower rates of Impella-related BARC type 3 or 5 bleeding and in-hospital death. Prospective multicenter studies should evaluate the reproducibility and effectiveness of standardized pathways extending from device insertion through ICU care.

## Supplementary Information

Additional file 1.

## Data Availability

No datasets were generated or analyzed during the current study.

## Abbreviations

AMI: Acute myocardial infarction
AMI-CS: Acute myocardial infarction-related cardiogenic shock
BARC: Bleeding Academic Research Consortium
CABG: Coronary artery bypass grafting
CHDF: Continuous hemodiafiltration
CI: Confidence interval
CS: Cardiogenic shock
CVP: Central venous pressure
ECMO: Extracorporeal membrane oxygenation
ECPELLA: Combined Impella and ECMO support
ECPR: Extracorporeal cardiopulmonary resuscitation
eGFR: Estimated glomerular filtration rate
HR: Hazard ratio
IABP: Intra-aortic balloon pump
ICU: Intensive care unit
J-PVAD: Japanese Registry for Percutaneous Ventricular Assist Device
LVEF: Left ventricular ejection fraction
MAP: Mean arterial pressure
MCS: Mechanical circulatory support
OW: Overlap weighting
PCCS: Postcardiotomy cardiogenic shock
PCI: Percutaneous coronary intervention
PDE3: Phosphodiesterase type 3
RBC: Red blood cell
rSO₂: Regional oxygen saturation
RV: Right ventricular
SCAI: Society for Cardiovascular Angiography and Interventions
SIM: Standardized Impella-specific management
SMD: Standardized mean difference
VA-ECMO: Venoarterial extracorporeal membrane oxygenation
