# Supplementary material for: Implementation of a standardized Impella-specific management pathway in cardiogenic shock
Source: J Intensive Care. 2026 Sep 10;14:101. doi: 10.1186/s40560-026-00937-9 (PMC13560191; doi:10.1186/s40560-026-00937-9)
Supplement: Supplementary file 1 [file 40560_2026_937_MOESM1_ESM.docx]

**Supplementary Material**

Implementation of a standardized Impella-specific management pathway in cardiogenic shock: a before-and-after study

**Supplementary Table 1. Implementation timeline and patient-level uptake of standardized Impella-specific management components**

| SIM component | Implementation date | Patients exposed within post-introduction cohort, n/N (%) | Patient-level uptake / implementation indicator | |
| --- | --- | --- | --- | --- |
| Education and implementation infrastructure | June 26, 2023 | 102/102 (100%) | Institutional-level intervention; no meaningful binary patient-level uptake measure. |  |
| Access-site and limb-perfusion management | June 26, 2023 | 102/102 (100%) | 16-Fr sheath use and standardized access/limb-perfusion management. Use of the 16-Fr sheath was 80.2% in the post-introduction group. |  |
| Peri-implant strategy | March 12, 2024 | 86/102 (84.3%) | Pre-PCI Impella use among eligible patients with AMI undergoing PCI was used as the patient-level practice indicator (76.2% in the post-introduction group). |  |
| Hemodynamic optimization | March 12, 2024 | 86/102 (84.3%) | This was a protocol-level intervention rather than a binary patient-level treatment. Implementation was assessed using patient-level process measures, including time outside the MAP target range and cumulative time with CVP ≥10 mmHg. |  |
| Quantitative plasma-free hemoglobin surveillance | April 14, 2024 | 84/102 (82.4%) | Surveillance was documented in 64/84 (76.2%) patients treated after introduction. Uptake increased from 18/34 (52.9%) before full implementation to 46/50 (92.0%) during full implementation. |  |
| Electronic medical record order set | January 18, 2025 | 57/102 (55.9%) | The order set was used in all 57 patients (100%) treated after its implementation. |  |
| Continuous lower-limb rSO₂ monitoring | April 1, 2025 | 50/102 (49.0%) | Monitoring was performed in 50/50 (100%) patients treated during the full-implementation phase. |  |

For components not appropriately represented by a binary patient-level adherence measure, the corresponding institutional or physiological implementation indicator is shown.

AMI, acute myocardial infarction; CVP, central venous pressure; MAP, mean arterial pressure; PCI, percutaneous coronary intervention; PFHb, plasma-free hemoglobin; rSO₂, regional oxygen saturation; SIM, standardized Impella-specific management.

**Supplementary Table 2. Components, Implementation Details, and Rationale for Standardized Impella-Specific Management**

| **Domain** | **SIM component** | **Operational details and triggers** | **Clinical objective and rationale** |
| --- | --- | --- | --- |
| **Education and implementation infrastructure** | **Multidisciplinary education and an Impella-specific electronic medical record order set** | Educational sessions and the Impella-specific order set established a shared approach to considering pre-PCI Impella support, evaluating suction or inadequate flow, managing suspected right ventricular dysfunction, and recognizing inadequate circulatory support. Education also covered reassessment and consultation about advanced MCS, including Impella 5.5. The order set specified monitoring frequency, hemodynamic targets, notification and reassessment triggers, and treatment options. Device selection and treatment intensity remained individualized. | Reduce practice variation, improve communication during transfer from the catheterization laboratory to the ICU, and prompt timely recognition and discussion of inadequate support. |
| **Peri-implant strategy** | **Structured assessment of Impella timing before PCI in selected patients with AMI-CS** | Training emphasized early identification of patients with AMI-CS, extensive jeopardized myocardium, systemic hypoperfusion, and/or congestion in whom Impella before PCI might be feasible. The decision remained individualized according to resuscitation status, coronary anatomy, and the urgency of revascularization. | Pre-PCI support was considered to establish left ventricular unloading and forward flow before revascularization, support hemodynamics during PCI, and avoid rescue implantation after further shock progression. |
|  | **Systemic perfusion checkpoint before leaving the catheterization laboratory** | Systemic perfusion was reassessed after Impella implantation using arterial blood gas analysis, serial lactate levels, blood pressure, oxygenation, urine output when available, effective Impella flow, suction events, echocardiography, and invasive hemodynamics when available. Persistent hypoperfusion, inadequate effective flow, or severe hypoxemia despite optimization prompted consideration of concomitant ECMO or another advanced MCS configuration. | Because Impella insertion alone does not ensure adequate systemic support, the checkpoint was used to identify patients who might require concomitant ECMO or another MCS configuration before ICU transfer. |
|  | **Lower-limb perfusion checkpoint before ICU transfer** | Lower-limb ischemia risk was assessed before transfer from the catheterization laboratory using distal pulses, skin temperature and color, capillary refill, and Doppler ultrasonography or angiography. If impaired limb perfusion was suspected, corrective measures, including antegrade 4-Fr perfusion-sheath placement, were considered before ICU transfer. | Identify access-related arterial obstruction before limb ischemia became prolonged or clinically overt. |
| **Access-site and limb-perfusion management** | **Non–peel-away 16-Fr sheath for femoral Impella CP support** | When clinically feasible, the manufacturer-supplied 14-Fr peel-away sheath was replaced with a non–peel-away 16-Fr sheath. The Impella catheter and repositioning sheath were maintained within the external sheath throughout support. | The 16-Fr sheath was used to improve catheter and sheath stability and limit peri-catheter leakage caused by displacement or incomplete sealing of the repositioning sheath. |
|  | **Continuous side-arm sheath flushing** | The side arm of the 16-Fr sheath was continuously flushed with 500 mL of saline containing 4,000 U of unfractionated heparin at 20 mL/h throughout Impella support and until sheath removal. | Continuous flushing was used to maintain sheath patency and limit thrombus formation within the sheath and related ischemic complications. |

**Supplementary Table 2. Continued**

| **Domain** | **SIM component** | **Operational details and triggers** | **Clinical objective and rationale** |
| --- | --- | --- | --- |
| **Access-site and limb-perfusion management** | **Continuous lower-limb rSO₂ monitoring** | Lower-limb rSO₂ was monitored continuously during femoral Impella support. An absolute rSO₂ value <40% or a relative decrease ≥20% from baseline prompted focused reassessment using physical examination and, when indicated, Doppler ultrasonography or angiography. | Continuous rSO₂ monitoring could detect changes between clinical examinations and prompt earlier assessment. The threshold triggered confirmation by clinical examination or imaging; it was not used alone to diagnose ischemia or mandate intervention. |
| **Hemolysis surveillance and management** | **Daily quantitative plasma-free hemoglobin monitoring and cause-directed reassessment** | Plasma-free hemoglobin was measured once daily during active Impella support, with additional measurement when clinically indicated. When plasma-free hemoglobin increased, device position, support level, suction events, left ventricular filling, preload status, right ventricular function, right-sided congestion, and overall hemodynamic conditions were reassessed and corrected when possible. | Routine measurement allowed earlier detection of hemolysis and reassessment of modifiable mechanical or hemodynamic causes before overt organ injury. |
|  | **Protocolized adjunctive haptoglobin administration** | Haptoglobin was administered according to plasma-free hemoglobin levels: 2,000 U for levels of 20 to <40 mg/dL and 4,000 U for levels ≥40 mg/dL. Administration was performed alongside investigation and correction of the underlying cause of hemolysis. | Haptoglobin was used to bind circulating cell-free hemoglobin and potentially limit tissue exposure to heme pigment. It supplemented, but did not replace, correction of device malposition, suction, inadequate preload, or other causes of hemolysis. |
| **Hemodynamic optimization** | **Trigger-based assessment and selective right ventricular/pulmonary vascular support** | Suction events, inadequate effective Impella flow, suspected right ventricular dysfunction, or a pulmonary artery pulsatility index <1.0 prompted reassessment of device position, volume status, bleeding, tamponade, congestion, and right ventricular function. Diastolic pulmonary artery pressure was also used as a practical surrogate for left-sided filling pressure during this hemodynamic reassessment. When impaired right ventricular output or excessive pulmonary vascular load was considered contributory, milrinone and/or inhaled nitric oxide were predefined options in the order set but were selected on an individual basis. | The assessment distinguished mechanical or preload-related suction from right ventricular failure. Milrinone or inhaled nitric oxide was considered when greater right ventricular output and pulmonary flow might improve left ventricular filling and effective Impella support. |
|  | **CVP-guided congestion and preload management** | CVP was reviewed serially and was generally targeted at <10 mmHg when clinically feasible, while preserving sufficient preload for stable Impella flow and avoiding suction events. CVP trends were interpreted together with effective device flow, lactate levels, urine output, echocardiography, and pulmonary artery catheter data to guide fluid administration, diuresis, fluid removal, and hemodynamic support. | Balance adequate preload for stable Impella flow against excessive right-sided filling pressure and systemic venous congestion. |
|  | **Blood pressure control and avoidance of excessive afterload** | Mean arterial pressure was targeted at 55–85 mmHg. Hypotension was treated according to systemic perfusion, whereas excessive afterload was managed when appropriate, including with vasodilator therapy. Blood pressure targets were interpreted together with lactate levels, urine output, effective Impella flow, and overall hemodynamic status. | The lower limit was chosen to preserve systemic and coronary perfusion, while the upper limit was intended to avoid excessive afterload that could reduce Impella flow and left ventricular unloading. |

Only the monitoring and reassessment framework was standardized; treatment remained individualized. Pre-PCI Impella, milrinone, inhaled nitric oxide, and advanced MCS were selected according to the evolving physiology and clinical context. Thresholds triggered further assessment and were not used alone as diagnostic or treatment criteria.

The systemic antithrombotic strategy was not otherwise changed after SIM implementation.

AMI-CS = acute myocardial infarction-related cardiogenic shock; CVP = central venous pressure; ECMO = extracorporeal membrane oxygenation; ICU = intensive care unit; LV = left ventricular; MCS = mechanical circulatory support; PAPi = pulmonary artery pulsatility index; PCI = percutaneous coronary intervention; rSO₂ = regional oxygen saturation; RV = right ventricular; SIM = standardized Impella-specific management.

**Supplementary Table 3. Baseline Characteristics Across the Three Implementation Phases**

| **Characteristic** | **Pre-implementation period (N=88)** | **Implementation phase (N=52)** | **Full-implementation phase (N=50)** | **Overall P value** |
| --- | --- | --- | --- | --- |
| **Demographics** | | | | |
| Age, years | 68.8 [58.2, 76.8] | 71.1 [60.4, 76.4] | 73.6 [63.0, 78.9] | 0.46 |
| Male sex | 67/88 (76.1%) | 42/52 (80.8%) | 39/50 (78.0%) | 0.82 |
| **Medical history** | | | | |
| Prior heart failure | 24/88 (27.3%) | 8/52 (15.4%) | 14/50 (28.0%) | 0.22 |
| Atrial fibrillation | 21/88 (23.9%) | 3/52 (5.8%) | 10/50 (20.0%) | 0.02 |
| Maintenance hemodialysis | 14/88 (15.9%) | 2/52 (3.8%) | 4/50 (8.0%) | 0.06 |
| Prior PCI or CABG | 28/88 (31.8%) | 6/52 (11.5%) | 4/50 (8.0%) | <0.001 |
| **Shock etiology, n (%)** | | | | |
| Acute myocardial infarction | 47/88 (53.4%) | 36/52 (69.2%) | 28/50 (56.0%) | 0.02 |
| Fulminant myocarditis | 11/88 (12.5%) | 9/52 (17.3%) | 6/50 (12.0%) |  |
| Heart failure/arrhythmia | 15/88 (17.0%) | 5/52 (9.6%) | 14/50 (28.0%) |  |
| PCCS/procedure-related/mechanical | 15/88 (17.0%) | 2/52 (3.8%) | 2/50 (4.0%) |  |
| **Presentation and shock severity** | | | | |
| Out-of-hospital cardiac arrest | 12/88 (13.6%) | 9/52 (17.3%) | 12/50 (24.0%) | 0.30 |
| In-hospital cardiac arrest | 26/88 (29.5%) | 10/52 (19.2%) | 13/50 (26.0%) | 0.40 |
| ECPR | 22/88 (25.0%) | 11/52 (21.2%) | 11/50 (22.0%) | 0.76 |
| SCAI classification |  |  |  | 0.61 |
| SCAI stage C | 15/88 (17.0%) | 12/52 (23.1%) | 7/50 (14.0%) |  |
| SCAI stage D | 25/88 (28.4%) | 10/52 (19.2%) | 12/50 (24.0%) |  |
| SCAI stage E | 48/88 (54.5%) | 30/52 (57.7%) | 31/50 (62.0%) |  |
| Systolic blood pressure, mmHg | 73.2 ± 50.5 | 79.2 ± 54.3 | 72.6 ± 49.0 | 0.76 |
| Lactate, mmol/L | 6.0 ± 3.9 | 7.4 ± 4.5 | 6.0 ± 4.2 | 0.14 |
| eGFR, mL/min/1.73 m² | 40.5 ± 25.0 | 45.1 ± 22.2 | 40.0 ± 23.3 | 0.46 |
| Total bilirubin, mg/dL | 1.0 ± 0.7 | 0.8 ± 0.5 | 1.0 ± 0.8 | 0.39 |
| Pre-Impella LVEF, % | 22.7 ± 11.8 | 23.2 ± 10.3 | 24.4 ± 15.0 | 0.73 |
| Hemoglobin, g/dL | 12.3 ± 2.3 | 12.7 ± 2.3 | 11.9 ± 2.4 | 0.17 |
| Platelet count, 10⁴/µL | 20.6 ± 9.9 | 25.5 ± 12.6 | 25.2 ± 15.7 | 0.03 |
| **Mechanical circulatory support at time zero** | | | | |
| ECPELLA at Impella insertion | 55/88 (62.5%) | 22/52 (42.3%) | 23/50 (46.0%) | 0.04 |

Values are presented as mean ± standard deviation, median [interquartile range], or n (%). Overall P values were calculated using one-way analysis of variance or the Kruskal–Wallis test for continuous variables, as appropriate, and Pearson’s χ² test or Fisher’s exact test for categorical variables, as appropriate. P values are descriptive and were not adjusted for multiple comparisons.

CABG = coronary artery bypass grafting; ECPELLA = concomitant VA-ECMO and Impella at Impella insertion; ECPR = extracorporeal cardiopulmonary resuscitation; eGFR = estimated glomerular filtration rate; LVEF = left ventricular ejection fraction; PCI = percutaneous coronary intervention; SCAI = Society for Cardiovascular Angiography and Interventions.

**Supplementary Table 4. Sensitivity Analyses for All-Cause In-Hospital Mortality**

| **Analysis** | **N (pre/post or full)** | **HR (95% CI)** | **P value** |
| --- | --- | --- | --- |
| **Primary analysis** | | | |
| Primary overlap-weighted cause-specific Cox model | 190 (88/102) | 0.51 (0.32–0.84) | 0.009 |
| **Sensitivity analyses** | | | |
| restricted sensitivity cohort | 168 (73/95) | 0.52 (0.32–0.84) | 0.008 |
| Excluding patients treated with Impella 2.5 | 178 (76/102) | 0.47 (0.30–0.76) | 0.002 |
| Primary overlap-weighted model additionally adjusted for calendar time | 190 (88/102) | 0.40 (0.17–0.95) | 0.04 |
| Excluding the implementation phase: pre-implementation vs full-implementation | 138 (88/50) | 0.48 (0.25–0.93) | 0.03 |

Propensity scores and weights were re-estimated within each analytic cohort. Pre/post or full denotes the pre-implementation and post-introduction periods or full implementation. The source-defined restricted cohort excluded 22 atypical presentations. All 12 Impella 2.5 cases occurred in the pre-implementation period; this analysis is therefore exploratory.

CI = confidence interval; HR = hazard ratio.

**Supplementary Table 5.** **Calendar-Year Trends in Clinical Outcomes**

| **Calendar year** | **Patients, n** | **In-hospital death, n/N (%)** | **30-day mortality, n/N (%)** | **Impella-related BARC type 3 or 5 bleeding, n/N (%)** |
| --- | --- | --- | --- | --- |
| 2018 | 11 | 6/11 (54.5%) | 6/11 (54.5%) | 2/11 (18.2%) |
| 2019 | 16 | 7/16 (43.8%) | 7/16 (43.8%) | 1/16 (6.2%) |
| 2020 | 18 | 12/18 (66.7%) | 9/18 (50.0%) | 7/18 (38.9%) |
| 2021 | 12 | 7/12 (58.3%) | 6/12 (50.0%) | 4/12 (33.3%) |
| 2022 | 20 | 14/20 (70.0%) | 10/20 (50.0%) | 7/20 (35.0%) |
| 2023 | 24 | 8/24 (33.3%) | 7/24 (29.2%) | 9/24 (37.5%) |
| 2024 | 30 | 12/30 (40.0%) | 11/30 (36.7%) | 4/30 (13.3%) |
| 2025 | 35 | 13/35 (37.1%) | 12/35 (34.3%) | 4/35 (11.4%) |
| 2026 | 24 | 6/24 (25.0%) | 3/24 (12.5%) | 2/24 (8.3%) |

Values are presented as n/N (%) unless otherwise indicated. Calendar-year analyses were descriptive and independent of the SIM implementation-period definitions.

**Supplementary Figure 1. Covariate balance before and after overlap weighting.**

Points show the absolute standardized mean differences. The dashed line indicates the prespecified |SMD| threshold of 0.10. All displayed variables were included in the primary propensity-score model.
